# Immunological causes of obsessive-compulsive disorder: is it time for the concept of an “autoimmune OCD” subtype?

**DOI:** 10.1038/s41398-021-01700-4

**Published:** 2022-01-10

**Authors:** Dominique Endres, Thomas A. Pollak, Karl Bechter, Dominik Denzel, Karoline Pitsch, Kathrin Nickel, Kimon Runge, Benjamin Pankratz, David Klatzmann, Ryad Tamouza, Luc Mallet, Marion Leboyer, Harald Prüss, Ulrich Voderholzer, Janet L. Cunningham, ECNP Network Immuno-NeuroPsychiatry, Katharina Domschke, Ludger Tebartz van Elst, Miriam A. Schiele

**Affiliations:** 1 Section for Experimental Neuropsychiatry, Department of Psychiatry and Psychotherapy, Medical Center - University of Freiburg, Faculty of Medicine, University of Freiburg, Freiburg, Germany; 2 Department of Psychiatry and Psychotherapy, Medical Center - University of Freiburg, Faculty of Medicine, University of Freiburg, Freiburg, Germany; 3 Department of Psychosis Studies, Institute of Psychiatry, Psychology and Neuroscience, King’s College London, London, UK; 4 Department for Psychiatry and Psychotherapy II, Ulm University, Bezirkskrankenhaus Günzburg, Günzburg, Germany; 5 AP-HP, Hôpital Pitié-Salpêtrière, Biotherapy (CIC-BTi) and Inflammation-Immunopathology-Biotherapy Department (i2B), Paris, France; 6 Sorbonne Université, INSERM, Immunology-Immunopathology-Immunotherapy (i3), Paris, France; 7 Univ Paris Est Créteil, INSERM, IMRB, Translational Neuropsychiatry, AP-HP, DMU IMPACT, FHU ADAPT, Fondation FondaMental, Créteil, France; 8 Department of Neurology and Experimental Neurology, Charité - Universitätsmedizin Berlin, Berlin, Germany; 9 German Center for Neurodegenerative Diseases (DZNE) Berlin, Berlin, Germany; 10 Schoen Clinic Roseneck, Prien am Chiemsee, Germany; 11 Department of Psychiatry and Psychotherapy, University Hospital Munich, Munich, Germany; 12 Department of Neuroscience, Psychiatry, Uppsala University, Uppsala, Sweden; 13 Centre for Basics in Neuromodulation, Faculty of Medicine, University of Freiburg, Freiburg, Germany

**Keywords:** Diagnostic markers, Molecular neuroscience

## Abstract

Obsessive-compulsive disorder (OCD) is a highly disabling mental illness that can be divided into frequent primary and rarer organic secondary forms. Its association with secondary autoimmune triggers was introduced through the discovery of Pediatric Autoimmune Neuropsychiatric Disorder Associated with Streptococcal infection (PANDAS) and Pediatric Acute onset Neuropsychiatric Syndrome (PANS). Autoimmune encephalitis and systemic autoimmune diseases or other autoimmune brain diseases, such as multiple sclerosis, have also been reported to sometimes present with obsessive-compulsive symptoms (OCS). Subgroups of patients with OCD show elevated proinflammatory cytokines and autoantibodies against targets that include the basal ganglia. In this conceptual review paper, the clinical manifestations, pathophysiological considerations, diagnostic investigations, and treatment approaches of immune-related secondary OCD are summarized. The novel concept of “autoimmune OCD” is proposed for a small subgroup of OCD patients, and clinical signs based on the PANDAS/PANS criteria and from recent experience with autoimmune encephalitis and autoimmune psychosis are suggested. Red flag signs for “autoimmune OCD” could include (sub)acute onset, unusual age of onset, atypical presentation of OCS with neuropsychiatric features (e.g., disproportionate cognitive deficits) or accompanying neurological symptoms (e.g., movement disorders), autonomic dysfunction, treatment resistance, associations of symptom onset with infections such as group A streptococcus, comorbid autoimmune diseases or malignancies. Clinical investigations may also reveal alterations such as increased levels of anti-basal ganglia or dopamine receptor antibodies or inflammatory changes in the basal ganglia in neuroimaging. Based on these red flag signs, the criteria for a possible, probable, and definite autoimmune OCD subtype are proposed.

## Background

Obsessive-compulsive disorder (OCD) is a severe and common mental illness [1] with a lifetime prevalence of 1–3% in adults [2, 3]. Because of the underlying stigma, considerable delays are common before OCD is diagnosed and a treatment is initiated [4]. Affected patients suffer from agonizing irrational thoughts (obsessions) that lead to a strong emotional reaction, such as anxiety or disgust, and repeated excessive behavior (compulsions) to reduce this anxiety [1, 5, 6]. The most common compulsive thoughts concern contamination (in ~50% of patients), and the most common compulsive behaviors are ordering (in ~60%) and washing rituals (in ~50%; [7]). OCD onset follows a bimodal age distribution and typically first manifests in late childhood and early adolescence or again in early adulthood [8]. The fifth version of the Diagnostic and Statistical Manual of Mental Disorders (DSM-5) distinguishes primary and secondary organic OCD forms [9]. In the tenth version of the International Statistical Classification of Diseases and Related Health Problems (ICD-10) criteria, only primary OCD is mentioned (https://www.who.int/classifications/icd/en/GRNBOOK.pdf), but a symptom code for obsessive-compulsive behavior is also included as an alternative (R46.81). In primary, idiopathic forms of OCD, a multifactorial etiology is assumed, in which biological, psychological, and external factors interact (cf. [10]). The biological processes include alterations in brain connectivity, such as an imbalance of cortico-striato-thalamo-cortical circuits, dysregulation of serotonergic, glutamatergic, and dopaminergic neurotransmission, and genetic and epigenetic influences (e.g., [1, 11–17]). Psychological models focus on the cognitive components (e.g., assessment) and learning experiences (e.g., the reduction of unpleasant sensations through certain behavioral patterns) as the disease’s factors of development and maintenance (“cognitive-behavioral theory”; [18]. External factors include burdens such as critical life events or infections (e.g., [19–21]). According to current recommendations, the most effective therapy for OCD is a combination of psychotherapy and pharmacotherapy. Psychotherapy involves disorder-specific cognitive-behavioral therapy with exposure and response prevention. First-line pharmacotherapy is performed with serotonin reuptake inhibitors [6, 22, 23]. Nevertheless, up to half of all OCD patients do not benefit from this combination treatment in a clinically meaningful way [6], which has prompted efforts aimed at identifying diagnostic subgroups with specific pathogenic mechanisms that may mediate treatment resistance, as well as improving treatment options. In this line, a subgroup of patients may have a secondary form of OCD with an organic cause, which often appears to be autoimmune mediated [19, 24–26]. In the novel ICD-11 criteria, reflecting DSM-5 nosology, this aspect is taken into account by introducing the category “secondary OCD or related syndrome” (https://icd.who.int). However, neither DSM-5 nor ICD-11 criteria provide specific instructions regarding the detection and delimitation of secondary forms.

Therefore, the rationale behind this conceptual review paper was to analyze the body of evidence for a putative link between OCD and immune activation and to outline a structured clinical approach to detect and treat secondary, autoimmune forms of OCD that can be evaluated and refined over time. For this purpose, based on an expert discussion and a PubMed search (see Box 1), a focused literature review was performed.

Box 1 A Pubmed search was performed using the mentioned search terms. The resulting 586 papers (retrieved 31.12.2020) were screened for the preparation of this narrative literature review Search strategy: “((PANDAS AND OCD) OR (PANS AND OCD) OR (autoimmune AND OCD) OR (immunological AND OCD) OR (inflammation AND OCD) OR (encephalitis AND OCD) OR (PANDAS AND obsessive compulsive disorder) OR (PANS AND obsessive compulsive disorder) OR (autoimmune AND obsessive compulsive disorder) OR (immunological AND obsessive compulsive disorder) OR (inflammation AND obsessive compulsive disorder) OR (encephalitis AND obsessive compulsive disorder))”.

## OCD and immune activation

### OCD and PANDAS/PANS

It is an established concept that obsessive-compulsive symptoms (OCS) may be associated with Pediatric Autoimmune Neuropsychiatric Disorder Associated with Streptococcal infection (PANDAS; [27–30]), which is proposed to be caused by an infection with group A beta-hemolytic *Streptococcus pyogenes*. It is assumed that reactive autoimmune phenomena can develop after infections that cause PANDAS [31]. The concept of PANDAS was recently supported by a Danish population-based cohort study of 1,067,743 children, demonstrating that children with positive streptococcal test results had an increased risk for developing OCD [32]. Interestingly, first degree relatives of patients with OCD also appear to be more frequently affected by streptococcal infections [33]. Recently, the concept of PANDAS has been extended to pathogens other than streptococcus and other autoimmune causes, and the term pediatric acute neuropsychiatric syndrome (PANS) was introduced. In this context, other pathogens such as *Mycoplasma pneumoniae*, *Borrelia burgdorferi*, *Borna disease virus*, and *Toxoplasmosis gondii* have also been identified as possible causes of immune-related PANS [19, 34]. Whether immune-mediated secondary OCD could also develop as a consequence of COVID-19 poses a highly relevant research question to be elucidated in the near future [35, 36]. The first studies of their kind have demonstrated infection-triggered neuronal antibody production against various antigens in COVID-19 patients who were presenting with unexplained neurological symptoms [37].

### Other immunological causes of OCD

**Fig. 1:**
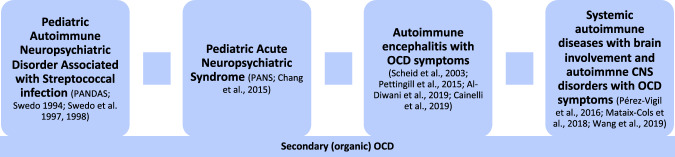
Autoimmune mediated secondary (organic) obsessive-compulsive syndromes [27–29, 34, 38–41, 44, 47, 48]. Abbreviations: CNS, central nervous system; OCD, obsessive-compulsive disorder.

Secondary OCD can also occur in the context of autoimmune encephalitis (AE; [38–42]) and is associated with established autoimmune diseases of the central nervous system (CNS), such as multiple sclerosis [43]. OCD syndromes can also occur in the context of systematic autoimmune diseases [44–49]. A nationwide study from Taiwan of 63,165 patients with a history of autoimmune disorders and 315,825 controls showed a higher incidence of OCD in patients with previous autoimmune disorders, especially in patients with systemic lupus erythematosus, dermatomyositis, and Sjögren’s syndrome [48]. Another nationwide study from Sweden in 30,082 patients with OCD demonstrated a significant correlation with different autoimmune disorders: Individuals with OCD had a 43% increased risk of any autoimmune disorders [47]. The different types of secondary, autoimmune OCD are summarized in Fig. 1.

Finally, immunological alterations could also be identified in patients with most likely primary forms of OCD; however, the results may be influenced by the prevalence of currently unrecognized secondary forms of OCD in some populations. Several genetic, cytokine, or antibody studies have suggested the involvement of immunological mechanisms (e.g., [6, 50, 51]). Table 1 gives an overview of the alterations in proinflammatory cytokines and antibodies found in patients with OCD. These observations are also supported by subclinical inflammatory changes in the blood and changes in the microbiome of patients with OCD [52, 53]. However, these observations are still inconclusive; for example, one meta-analysis on cytokine changes in serum also revealed no significant differences compared with the controls [54].

**Table 1:** Evidence of immunological causes in patient cohorts with assumed primary forms of obsessive-compulsive disorders (extracted from [26]).

Pro-inflammatory cytokines	
Gray and Bloch, 2012 (Meta-analysis) [55]	• Decreased IL-1β levels and decreased TNFα levels in non-depressed patients with OCD
	• Increased IL-6 levels in adult (medication-free) patients with OCD
Jiang et al., 2018 (Meta-analysis) [56]	• TNF-α-238G/A gene polymorphism could lead to a decreased risk of OCD susceptibility
Cosco et al., 2019 (Systematic review and meta-analysis) [54]	• No alterations in different immune mediators (IL-1β, IL-4, IL-6, IL-10, TNF-α, and interferon-γ).
Antibodies/Infections	
Pearlman et al., 2014 (Meta-analysis) [57]	• High frequency of anti-basal ganglia antibodies
Sutterland et al., 2015 (Meta-analysis) [58]	• Association with toxoplasma infection
Lamothe et al., 2018 (Systematic review) [26]	• High frequency anti-streptolysin O, anti-streptokinase, and anti-DNase B antibodies (e.g., [59])
	• Anti-dopamine (D1/2) receptor antibodies and anti-lysoganglioside antibodies are more frequent in patients with PANDAS and obsessive-compulsive symptoms (e.g., [60])
	• Anti-enolase antibodies are frequent [61]
	• More CSF anti-brain antibody binding to basal ganglia and thalamus was described [62]
	• Herpes IgG antibodies were more frequent in CSF of patients compared with controls [63]

*CSF* cerebrospinal fluid, *IgG* immunoglobulin G, *IL* interleukin, *OCD* obsessive-compulsive disorder, *PANDAS* Pediatric Autoimmune Neuropsychiatric Disorder Associated with Streptococcal infection, *TNF* tumor necrosis factor.

## Clinical presentation of patients with possible immune-related OCD

### Diagnostic criteria of PANDAS

The PANDAS consensus criteria were developed by Swedo and colleagues [27–29] and describe a neuropsychiatric syndrome with the presence of OCD or tic disorder and a temporal association of symptom onset with group A streptococcal infection. Up to now, reports of similar cases in adulthood are rare [64–67]. PANDAS can be diagnosed with all of the following symptoms (1–5) present:

1. Presence of OCD or a tic disorder,
2. Prepubertal symptom onset,
3. Acute symptom onset and episodic (relapsing-remitting) course,
4. Temporal association between Group A streptococcal infection and symptom onset/exacerbations,
5. Association with neurological signs (motor hyperactivity or choreiform movements; [34, 68]).

### Diagnostic criteria of PANS

The PANS criteria were developed at the 2013 PANS Consensus Conference [34]. They represent an extension of the PANDAS criteria. PANS can also be diagnosed in the presence of other neurotropic pathogens besides streptococci or in the context of other autoimmune diseases and without the presence of neurological symptoms [69, 70]. The following criteria (1–3) must be met:

1. Abrupt, dramatic onset of OCD or severely restricted food intake.
2. Concurrent presence of additional neuropsychiatric symptoms (with similarly severe and acute onset), from at least two of the following seven categories:

- ○ Anxiety,
- ○ Emotional lability and/or depression,
- ○ Irritability, aggression, and/or severely oppositional behaviors,
- ○ Behavioral (developmental) regression,
- ○ Deterioration in school performance (related to attention-deficit-hyperactivity disorder-like symptoms, memory deficits, cognitive changes),
- ○ Sensory or motor signs,
- ○ Somatic alterations and symptoms, including sleep disturbances, enuresis, or in urinary frequency.

3. Symptoms are not better explained by a known neurologic or medical disorder, such as Sydenham chorea, systemic lupus erythematosus, Tourette disorder or others [34].

The PANS criteria are quite broad and, thus, “non-specific.” The rationale for omitting tics from the PANS criteria and adding eating disorders with severely restricted food intake (compared with the PANDAS criteria) contributes to the broadening of the concept. Thus, PANS criteria can only give indications of a possible autoimmune OCD syndrome, but can by no means prove it. Further research is required to establish the sensitivity and specificity of these criteria vis-à-vis an immunotherapy-responsive OCD syndrome, although notable trials of immunotherapy in this group have been negative or inconclusive (summarized in [71]), potentially suggesting a need for modification of the criteria.

### Possible signs of immune-related psychiatric syndromes in general

Even established AE may present with predominant or isolated psychiatric disorders [42]. In this context, OCS may also occur [38–42, 72]. Associations have been described for patients with anti-NMDA-R, anti-GABA_A_, anti-dopamine (D1/D2), anti-basal ganglia, and anti-Ma2/CV2 antibodies [38–41, 57, 60, 72]. In 2016, international consensus criteria for a possible AE were defined for the first time [73]. In 2017, yellow and red flag symptoms for identifying AE in psychiatric patients were published [74]. In 2020, international consensus criteria for autoimmune psychosis (AP) were published for the first time [75]. Table 2 gives an overview of these previous concepts.

**Table 2:** Consensus criteria for autoimmune encephalitis and autoimmune psychosis, as well as red and yellow flag symptoms for autoimmune encephalitis in psychiatric patients [73–75].

Consensus criteria for possible autoimmune encephalitis [73]	Red- and yellow-flag symptoms for autoimmune encephalitis [74]	Consensus criteria for autoimmune psychosis [75]
1. Subacute onset (rapid progression of less than 3 months) of working memory deficits, altered mental status, or psychiatric symptoms	Yellow flag symptoms/findings:	Possible autoimmune psychosis:
2. At least one of the following:	◦ Decreased levels of consciousness	Psychotic symptoms of abrupt onset (rapid progression of <3 months) with at least one of the following:
◦ New focal neurological findings	◦ Abnormal postures or movements (orofacial, limb dyskinesia)	◦ Currently or recently diagnosed with a tumor
◦ Seizures	◦ Autonomic instability	◦ Movement disorder (catatonia or dyskinesia)
◦ CSF pleocytosis	◦ Focal neurological deficits	◦ Adverse response to antipsychotics, raising suspicion of neuroleptic malignant syndrome
◦ MRI suggestive of encephalitis	◦ Aphasia or dysarthria	◦ Severe or disproportionate cognitive dysfunction
3. Exclusion of alternative causes	◦ Rapid progression (despite therapy)	◦ A decreased level of consciousness
	◦ Hyponatremia	◦ Seizures
	◦ Catatonia	◦ Autonomic dysfunction (abnormal or unexpectedly fluctuant blood pressure, temperature, or heart rate)
	◦ Headache	Probable autoimmune psychosis:
	◦ Other autoimmune diseases (e.g., thyroiditis)	Current psychotic symptoms of abrupt onset (rapid progression of <3 months) with at least one of the seven clinical criteria listed above
	Red flag symptoms/findings:	*and at least one of the following:*
		◦ CSF pleocytosis
		◦ Bilateral brain abnormalities on T2-weighted FLAIR MRI highly restricted to the medial temporal lobes
	◦ CSF pleocytosis or CSF-specific oligoclonal bands	*or two of the following:*
	◦ Seizures/faciobrachial dystonic seizures	◦ “Encephalopathic” EEG changes (i.e., spikes, spike-wave activity, or rhythmic slowing, focal changes, or extreme delta brush)
	◦ Suspected malignant neuroleptic syndrome	◦ CSF-specific oligoclonal bands or increased IgG index
	◦ MRI abnormalities (mesiotemporal hyperintensities, atrophy pattern)	◦ The presence of a serum neuronal antibody detected by cell-based assay
		◦ After exclusion of alternative diagnoses.
	◦ EEG alterations (slowing, epileptic activity or extreme delta brush)	Definite autoimmune psychosis:
		Probable autoimmune psychosis with
		◦ IgG class neuronal antibodies in CSF.

*CSF* cerebrospinal fluid, *EEG* electroencephalography, *FLAIR* fluid-attenuated inversion recovery, *IgG* immunoglobulin G, *MRI* magnetic resonance imaging.

### Signs of potential autoimmune OCD

Based on the above-mentioned criteria for PANDAS/PANS, the experiences with AE/AP, the presently available literature on immune-related OCDs, and expert consensus, we propose preliminary “red flag signs” for potential autoimmune OCD (see Box 2).

Box 2 Red flag symptoms for potential autoimmune obsessive-compulsive disorder *Development of OCD weeks to months after an infection. **”Non-specific” markers. Abbreviations: ANA, antinuclear antibodies; CSF, cerebrospinal fluid; dsDNA, double strand deoxyribonucleic acid; EEG, electroencephalography; FDG-PET, [^18F^]fluorodeoxyglucose positron emission tomography; FLAIR, fluid-attenuated inversion recovery; GFAP, glial fibrillary acidic protein; IgG, immunoglobulin G; MRI, magnetic resonance imaging; NfL, neurofilament light chain; NMDA-R, N-methyl-d-aspartate receptor; OCD, obsessive-compulsive disorder; R, receptor.

**Table:** 

• (Sub)acute onset of OCD (< 3 months)
• Treatment resistance despite guideline-based therapy
• Atypical age of onset (early childhood or later adulthood)
• Atypical presentation of OCD (e.g., combination with severe hypersomnia or loss of function due to disproportionate cognitive deficits)
• Accompanying neurological signs:
➢ Movement disorder (catatonia, choreiform movements, dyskinesia, etc.)
➢ Focal neurological deficits
➢ New seizures
➢ New headache
• Autonomic dysfunction (e.g., hyperthermia, tachycardia, fluctuating blood pressure)
• Adverse response to antipsychotics (especially if malignant neuroleptic syndrome is suspected)
• Temporal association* of OCD onset with infections (such as Group A streptococcal infection)
• Comorbid autoimmune diseases (such as multiple sclerosis or systemic lupus erythematosus)
• Comorbid malignancies (such as ovarian teratoma)
• Suspicious alterations in diagnostic investigations:
➢ Serum: Neuronal autoantibodies (e.g., against NMDA-R, basal-ganglia, dopamine 1/2-R), “potentially neuronal” antibodies (e.g., ANAs against dsDNA), streptococcal antibodies
➢ EEG: Signs of encephalopathy such as spike-wave activity or intermittent slowing
➢ MRI: Basal ganglia/mesiotemporal hyperintensities, inflammatory lesions
➢ FDG-PET: Encephalitic patterns with disturbed metabolism in basal-ganglia, and along cortical or in temporal regions
➢ CSF: CSF-pleocytosis, CSF-specific oligoclonal bands, detection of (neuronal) autoantibodies, increased antibody indices, elevated damage markers (such as NfL, GFAP, tau**)

## Pathophysiological considerations

### The PANDAS/PANS model

Infections with ß-hemolytic streptococcus, such as *Streptococcus pyogenes*, most frequently lead to localized diseases of the throat, resulting in tonsillopharyngitis. Subsequent complications of streptococcal infections may include acute rheumatic fever, acute glomerulonephritis, chorea minor, and PANDAS. OCS in patients with PANDAS usually first emerges with a latency of a few weeks (https://www.rki.de/DE/Content/Infekt/EpidBull/Merkblaetter/Ratgeber_Streptococcus_pyogenes.html; accessed 4 August 2021). Infection with *Streptococcus pyogenes* and the subsequent production of anti-streptococcal antibodies (anti-streptolysin O and anti-DNase B antibodies; [76]) could trigger an autoimmune response via molecular mimicry, a process by which the host antibodies directed against *Streptococcus pyogenes* cross-react with different basal ganglia epitopes [77, 78]. In light of the evidence that OCD is associated with relevant dysfunction of the basal ganglia [1, 17], it seems plausible that such antibodies against different basal ganglia epitopes may induce OCS. Along these lines, findings from animal models support the hypothesis that streptococcal infections can induce OCS [79]. However, there is no direct demonstration that the sole injection of such antibodies can cause OCD. In patients with PANDAS, reactive anti-D1/D2R, anti-basal ganglia, anti-lysoganglioside, and anti-tubulin antibodies, along with the antibody-mediated activation of calcium calmodulin-dependent protein kinase II (CaMKII), have been detected [57, 60, 78, 80, 81]. The activation of CaMKII could alter dopamine release [82]. So far, the effects of anti-D1/D2R antibodies are best understood as follows: in vitro, they lead to the internalization of dopamine receptors, potentially resulting in vivo in basal ganglia encephalitis and hyperintense signals in the basal ganglia [83]. Two recently published studies have demonstrated that immunoglobulin (Ig) G antibodies in the serum of patients with PANDAS also appear to bind specifically to cholinergic interneurons in the striatum [78, 84]. A dysfunctional blood brain barrier (BBB) function could theoretically allow antibodies to pass, and evidence for this has been shown in animal models and patients with OCD [85, 86]. In a recently developed mouse model, repeated group A streptococcal intranasal challenge promoted the migration of Th17 cells with group A streptococcal specificity into the brain, BBB disturbance, serum IgG deposition in the brain, microglial activation, and loss of excitatory synaptic proteins [87].

### Other autoimmune causes of OCD

The established neuronal antibodies can be divided into paraneoplastic antibodies against intracellular antigens (such as anti-Ma2 or anti-CV2 antibodies), which are usually only epiphenomena for the immune process, and neuronal antibodies against cell-surface antigens (such as anti-NMDA-R or anti-D1/D2R antibodies), which can occur without malignancies and have direct pathophysiological relevance because of their localization on the cell surface [83]. The pathophysiology of other autoimmune CNS diseases or systemic autoimmune diseases with brain involvement has been detailed elsewhere (e.g., [73, 83, 88]). It can be assumed that for these diseases, OCS are especially likely to occur in patients who experience an inflammatory reaction in structures along the cortico-striato-thalamo-cortical circuits, especially given their established involvement in primary OCD (cf. [1, 11, 17]). Besides primary autoimmune mechanisms, mild neuroinflammation may be caused secondarily by infections with various neurotropic viruses or other infectious agents, such as Lyme borreliosis. The existence, nature, and specificity of a T cell–mediated immune response that would be involved in OCD has not been studied so far. The temporal relationship between an infection and development of the symptoms raises the possibility that T cells with antimicrobial T cell receptors (TCRs) that are stimulated by the infection might attack the CNS [89]. Because regulatory T cells (Treg) play a major role in maintaining immune homeostasis, controlling inflammation, and protecting the body from autoimmune diseases [90], their stimulation offers therapeutic opportunities for many autoimmune diseases [91, 92], including AE [93].

The following causes of mild neuroinflammation, which may lead to inflammatory forms of OCD, are currently distinguished [31]:

1. A combination of infectious and autoimmune processes (e.g., in PANDAS/PANS or in patients with anti-NMDA-R encephalitis after Herpes simplex encephalitis; [37, 94–96].
2. Autoimmune mechanisms (e.g., in patients with neuronal autoantibodies or multiple sclerosis).
3. Infectious cause (i.e., low-grade “encephalitis” caused by various neurotropic viruses, etc.; [31]).

### HLA, a possible immunogenetic substratum of OCD

The “infection-inflammation-immune activation” triade observed in OCD suggests an involvement of the human leukocyte antigen (HLA) system, which is a central player in both innate and adaptive immune responses. In addition, the HLA system is now well recognized to be at the crossroads between neurodevelopmental processes and common major psychiatric conditions [97]. These characteristics have prompted earlier studies that—albeit showing an association with certain secondary causes of OCD like Sydenham’s chorea, which is the neurological manifestation of rheumatoid fever—did not provide consistent data, which may however be partly because of study design pitfalls including small sample sizes and/or clinical heterogeneity [98, 99]. Nevertheless, at a large-scale level, a recent exon-based genome-wide association study (GWAS) in OCD patients from Spain showed strong associations with two different regions of the HLA-hosting major histocompatibility complex (MHC; [100]) on the one hand, and a shared polygenic risk with schizophrenia, the most associated psychiatric condition with the MHC region [101], on the other hand. Surprisingly, the two identified regions did not correspond to the ones associated with schizophrenia risk, highlighting the complexity of MHC/HLA involvement in the mental disorders that are afflicted by the relative absence of information concerning the potential weight of the classical HLA. Illustrating this notion, a very recent study exploring the relationship between early-onset OCD and HLA-class II genetic diversity [51] reported an association with the autoimmune cornerstone *HLA-DRB1-04*, which is well known to be involved in the development of classical inflammatory and autoimmune disorders, such as type 1 diabetes [102] or rheumatoid arthritis [103]. In the latter case, it is worth noting that at the allelic level, the observed associations include both HLA-DRB1*01, *04, and *05 variants. These variants all bear amino acids defining the prototypic rheumatoid arthritis-associated shared epitope [104]. They have also been demonstrated to be involved in the pathogenesis of autism spectrum disorders [105] that share some clinical features with OCD and which often occur comorbid with OCD [106]. Finally, also supporting an implication of HLA in OCD is the interplay between *streptococcal* infections and HLA alleles [107]. In light of these considerations, the HLA system may play a bigger role in OCD than previously believed, which will have to be elucidated in large, future studies examining the distribution of classical HLA alleles in OCD.

## Diagnostic investigations and typical findings

### Suggestions for the diagnosis of PANDAS/PANS

Clinical evaluations should include psychiatric and neurological evaluations, an assessment of somatic symptoms, infectious disease evaluation (streptococcal infection or associated [non]febrile infectious illnesses such as rhinosinusitis, pharyngitis, or bronchitis), assessment of symptoms and history that necessitate further evaluation of immune dysregulation (i.e., autoimmune disease, inflammatory disease) or an evaluation for immunodeficiency, and medical and family history [34]. Laboratory diagnostics in patients with suspected PANDAS/PANS should include the detection of underlying pathogens, various laboratory analyses, and additional investigations according to common consensus criteria [34] (see Supplemental Table 1).

### Diagnostic possibilities for the detection of autoimmune OCD in general

Based on studies in patients with PANDAS/PANS, as well as AE and AP, different blood analyses, electroencephalography (EEG), MRI, and CSF investigations may be useful to detect an autoimmune cause of OCD [34, 73, 75, 108, 109]. Administration of [^18F^]fluorodeoxyglucose positron emission tomography (FDG-PET) and/or 18-kDa translocator protein (TSPO-PET) may also be helpful in distinguishing between autoimmune and primary OCD [109–113]. The “Cunningham panel” is proposed to measure PANDAS/PANS-associated antibodies, including anti-D1/D2R, anti-β-tubulin, and anti-lysoganglioside antibodies, as well as CaMKII activity [114–116]. However, as a limitation, it must be mentioned that the “Cunningham panel” has not been validated in adults and has failed in tests of sensitivity or specificity in a previous study, so its clinical utility is currently questionable [117]. According to the experiences with AE/AP, confirmatory tests should be used for neuronal antibodies with established methods, such as fixed cell-based assays, live cell-based assays, immunoblots, and/or indirect immunofluorescence on murine brain sections [73, 75, 109, 118–121]. Table 3 gives an overview of potentially helpful diagnostic tests.

**Table 3:** Diagnostic investigations for the detection of autoimmune causes of obsessive-compulsive disorders and other psychiatric syndromes (compare with [34, 108, 121]).

Patient history	
Onset/course of OCD	• Acute onset (< 3 months)? • Treatment resistance or poor response to standard treatment according to OCD guidelines? • Neurological symptoms such as seizures or focal neurological signs? • Autonomic instability? Infectious prodromic symptoms? Systemic signs?
Family history	• Psychiatric/neurological/immunological/malignant disorders?
Comorbidity	• Psychiatric co-morbidity (Psychosis? Depression? Dementia? Autism? Tics?) • Peripheral or central nervous neurological disorders (Encephalitis? Multiple sclerosis?) • Systemic autoimmune disorders (Systemic lupus erythematosus? Sjögren’s Syndrome?) • Malignancies?
**Physical examination**	
Internal	• Tonsillopharyngitis? Heart involvement? Skin? Fever? Gastrointestinal symptoms? Changes in body weight? Etc.?
Neurological	• Movement disorders? Focal neurological signs? Kayser–Fleisher rings? Etc.?
**Blood analyses**	
Basic blood analyses	• Differential blood count • Electrolytes (sodium, potassium, calcium, magnesium) • Metabolic markers (Creatinine, CK, GOT, GPT, AP, γ-GT, lipase) • Thyroid hormones (TSH, free T3, free T4) • Coagulation (INR, Quick, PTT) • Caeruloplasmin and copper
Serologies or PCR analyses	• When suspected association to streptococcal infection: Anti-streptolysin O and anti-DNAse B antibodies (throat culture for Group A streptococcal infection) • Borrelia burgdorferi (Lyme disease), mycoplasma pneumonia, Influenza, Epstein Barr virus, Herpes simplex, varicella zoster, toxoplasmosis gondii, Borna Disease Virus etc.
Rheumatic/immunological screening	• CRP, IgG/IgA/IgM levels, immune fixation • CH50, C3, C4, C3d • Rheumatoid factor • ANA (dsDNA, ENA-differentiation), ANCA (MPO/PR3), antiphospholipid antibodies (lupus anticoagulant, anti-cardiolipin antibodies, anti-β2-glycoprotein I antibodies) • Anti-thyroid antibodies (TPO/TG/TRAK)
Neuronal antibodies	• Against well-characterized cell surface antigens: NMDA-R, AMPA-1/2-R, GABA_A_/_B_-R, LGI1, CASPR2 • Against well-characterized intracellular antigens: Yo, Hu, CV2/CRMP5, Ri, Ma1/2, SOX1, Tr, Zic4, GAD65, amphiphysin • When available: “Tissue tests” (indirect immunofluorescence on murine brain sections) for novel neuronal antibodies • “Suspected PANDAS antibodies”: Antibodies against basal ganglia or dopamine D1/D2 receptor, perhaps also antibodies directed against lysoganglioside, tubulin, as well as antibody produced activation of calcium calmodulin protein kinase II
**Urine**	
Instrument-based diagnostics	• Urine status/culture, drug screening, pregnancy test (for women)
EEG	• Resting state EEG including hyperventilation period, in special cases: “sleep EEG”
Brain MRI	• T1-weighted/MPRAGE, FLAIR, DWI sequences etc. (https://generate-net.de/generate-sops.html)
**Cerebrospinal fluid analyses**	
Routine analyses	• White blood cell count, total protein, albumin quotient, IgG index, OCBs in serum/CSF, lactate • Damage markers optionally (such as NfL, GFAP, tau; https://07525720-0688-4380-840d-0a4af942fef7.filesusr.com/ugd/92c932_af60b043468c4969b48f3c46bfc9b30f.pdf)
Antibody testing	• Against different well-characterized cell surface antigens: NMDA-R, AMPA-1/2-R, GABA_A/B_-R, LGI1, CASPR, basal ganglia, dopamine D1/D2 receptor • Against well-characterized intracellular antigens: Yo, Hu, CV2/CRMP5, Ri, Ma1/2, SOX1, Tr, Zic4, GAD65, amphiphysin • When available: “Tissue tests” for novel neuronal antibodies • Antibody indices: For neuronal antibodies or pathogens (e.g., for antibodies against Borrelia burgdorferi); MRZ-reaction
**Other investigations (for selected cases)**	
Psychometric/ neuropsychological testing	• Yale-Brown Obsessive-Compulsive Scale • Test battery for attentional performance and for other executive functions (https://generate-net.de/generate-sops.html)
Positron emission tomography (PET)	• FDG- or TSPO-PET
Screening for malignancies	• According to the tumor-specific guidelines (in patients with paraneoplastic antibodies)

*OCD* obsessive-compulsive disorder, *CK* creatine kinase, *GOT* glutamate oxaloacetate transaminase, *GPT* glutamate pyruvate transaminase, *AP* alkaline phosphatase, *γ-GT* gamma-glutamyltransferase, *TSH* thyroid-stimulating hormone, *T3* triiodothyronine, *T4* thyroxine, *INR* international normalized ratio, *PTT* partial thromboplastin time, *CRP* C-reactive protein, *Ig* immunoglobulin, *CH50* total hemolytic complement activity, *C3/C4/C3d* complement factors, *ANA* antinuclear antibody, *ENA* extractable nuclear antigens, *ANCA* anti-neutrophil cytoplasmic antibodies, *MPO* myeloperoxidase, *PR3* proteinase-3, *TPO* thyroid peroxidase, *TG* thyroglobulin, *TRAK* TSH receptor autoantibodies, *NMDA-R* N-methyl-d-aspartate type glutamate receptor, *AMPA-1/2-R* α-amino-3-hydroxy-5-methyl-4-isoxazolepropionic acid-1/2-receptor, *GABAA/B-R* γ-aminobutyric acid-A/B-receptor, *LGI1* leucine-rich, glioma inactivated 1, *CASPR2* contactin-associated protein-like 2, *Yo/Hu* initials of the first described patient, *CV2/CRMP5* collapsin response mediator protein 5, *Ri* initials of the first described patient, *SOX1* sry-like high mobility group Box 1, *Zic4* zinc-finger of the cerebellum protein, *GAD65* glutamate-decarboxylase 65kD, *PANDAS* Pediatric Autoimmune Neuropsychiatric Disorder Associated with Streptococcal infection, *EEG* electroencephalography, *MRI* magnetic resonance imaging, *MPRAGE* magnetization prepared - rapid gradient echo, *FLAIR* fluid-attenuated inversion recovery, *DWI* diffusion-weighted imaging, *OCBs* oligoclonal bands, *CSF* cerebrospinal fluid, *GFAP* glial fibrillary acidic protein, *NfL* neurofilament light chain, *FDG-PET* [18F]fluorodeoxyglucose positron emission tomography, *TSPO-PET* 18-kDa translocator protein positron emission tomography.

### Typical diagnostic findings

#### PANDAS

**Fig. 2:**
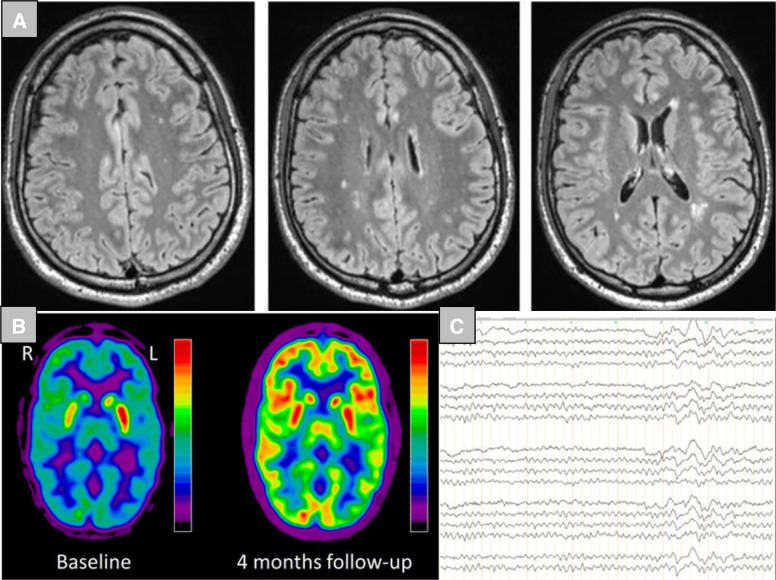
Exemplary findings suggestive of an autoimmune cause of obsessive-compulsive symptoms. **A** The magnetic resonance imaging (MRI) shows multiple chronic inflammatory white matter lesions using FLAIR sequences. The corresponding patient was 22 years old at the time of the MRI, was diagnosed as neuropsychiatric lupus erythematosus and suffered from an atypical OCD with abrupt onset and additional psychotic symptoms. Immunotherapy with steroids, methotrexate and hydroxychloroquine resulted in an impressive clinical improvement [46] (online available at: https://www.frontiersin.org/articles/10.3389/fpsyt.2019.00226/full, retrieved on 30 January 2021; the reproduction was allowed by the publisher according to the Creative Commons Attribution License [CC BY]). **B** The [^18F^]fluorodeoxyglucose positron emission tomography (FDG-PET) findings are from of an 18-year-old male patient with suspected PANDAS syndrome. Baseline images showed moderate to severe hypermetabolism of the left striatum. The cortex showed a hypometabolic signal. The metabolic changes disappeared completely after plasmapheresis treatment at 4 months follow-up [67] (online available at: https://bmcneurol.biomedcentral.com/articles/10.1186/s12883-018-1063-y, retrieved on 30 January 2021; the reproduction was allowed by the publisher according to the Creative Commons Attribution 4.0 International License). **C** Intermittent rhythmic generalized electroencephalography (EEG) slowing in 41-year-old female patient with Hashimoto encephalopathy [129] (online available at: https://www.frontiersin.org/articles/10.3389/fpsyt.2017.00064/full, retrieved on 30 January 2021; the reproduction was allowed by the publisher according to the Creative Commons Attribution License [CC BY]). This EEG phenomenon is frequently found in patients with autoimmune psychiatric syndromes [75].

When the medical history suggests an association with streptococcal infection, such as in PANDAS, streptococcal antibodies (i.e., anti-streptolysin O and anti-DNase B antibodies) may be detected in the blood. Additional cross-reactive neuronal (e.g., anti-basal ganglia) antibodies may be found in the serum and/or CSF, but the clinical significance of these has not been established. In severe cases, T2-weighted MRI images may show inflammatory changes in the basal ganglia or volumetric changes in basal ganglia structures [34, 122, 123]. In EEG studies, focal/generalized slowing or spike wave activity was detected in 16% of patients [34]. Casuistically, FDG-PET hypermetabolism has been described in the basal ganglia and hypometabolism along the cortex ([67]; Fig. 2). These can presumably turn into hypometabolic changes in prolonged courses of the disease (cf. [124]). Neuropsychological testing usually reveals executive dysfunction [125–127].

#### Autoimmune OCD in general

In other patients with autoimmune OCD, systemic (e.g., ANAs with dsDNA specificity) or neuronal antibodies (e.g., anti-Ma2 or NMDA-R antibodies) may be found in the blood and/or CSF. Typical MRI findings include lesions of the basal ganglia [128], (chronic) inflammatory lesions in patients with multiple sclerosis or mixed connective tissue disorders (Fig. 2; [46]), or mesiotemporal hyperintensities in patients with limbic encephalitis [83]. EEG may show signs of encephalopathy (Fig. 2; [129]). CSF alterations with intrathecal antibody production (increased antibody indices or intrathecal production of neuronal, e.g., anti-NMDA-R, antibodies) in combination with acute (increased CSF white blood cell count) or chronic inflammatory changes (e.g., increased IgG indices, focal Ig synthesis, oligoclonal bands) are possible indicators of autoimmune OCD forms. “Encephalitic” FDG-PET patterns include disturbed metabolism of basal ganglia/diffuse cortical structures or focal temporal structures [67, 130–132]. Studies using TSPO-PET have revealed microglia activation in the dorsal caudate, orbitofrontal cortex, thalamus, ventral striatum, and dorsal putamen [111].

## Therapeutic implications

### Treatment of PANDAS/PANS

**Fig. 3:**
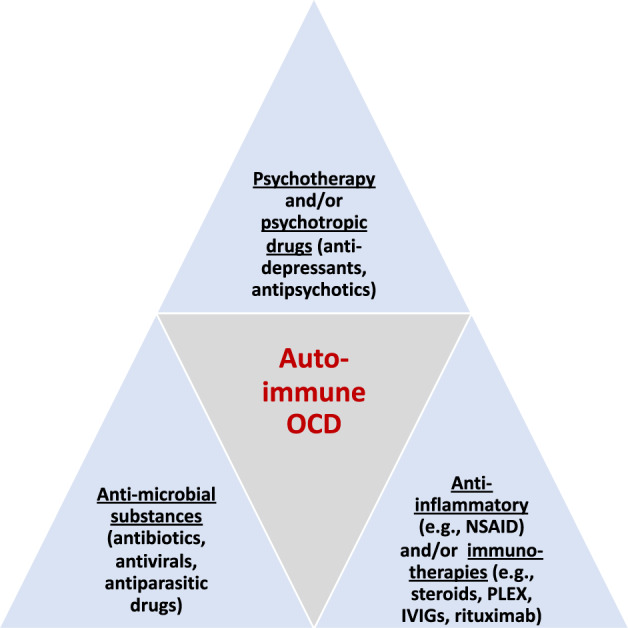
The three main components in the treatment of autoimmune obsessive-compulsive disorders (cf. [71, 73, 134–136]). Abbreviations: OCD, obsessive-compulsive disorder; IVIGs, intravenous immunoglobulins; NSAID, non-steroidal anti-inflammatory drug; PLEX, plasmapheresis.

As with primary OCD, psychotherapeutic and psychopharmacological (serotonin reuptake inhibitors, clomipramine, atypical antipsychotics) therapies constitute the standard treatment for PANDAS/PANS patients [133, 134]. For treatment-resistant patients, further treatment alternatives with anti-microbial substances that work against different pathogens and anti-inflammatory/immunotherapies that are primarily directed at reactive autoimmune processes are available (see Fig. 3). The current guidelines are based on expert recommendations, not on randomized controlled trials (RCTs; [71]). According to expert recommendations [134, 135], nonsteroidal anti-inflammatory drugs (NSAIDs) and/or short oral steroid bursts are suggested in cases of persistent symptoms after failure of classical psychotherapeutic and psychopharmacological treatment. For moderate to severe symptoms, steroids or IVIGs may be sufficient. For severe or chronic presentations, prolonged steroid trials or repeated high-dose steroids may be indicated. For patients with extreme impairment, therapeutic plasma exchange is the first-line therapy and could be applied alone or in combination with IVIGs, high-dose intravenous steroids, and/or rituximab [134, 136]. Antibiotic prophylaxis is suggested for children with PANDAS who have a severe form and/or recurrent courses of streptococcus-associated exacerbations or an initial manifestation ([135]; https://www.pandasppn.org/wp-content/uploads/PANDAS_Flow_Chart.pdf).

Sigra et al. (2018) systematically investigated the effects of antibiotics, steroids, intravenous immunoglobulins (IVIGs), plasma exchange, tonsillectomy, and NSAIDs in 240 patients with PANDAS/PANS [71] and found only four RCTs in children with PANDAS that have been conducted so far [137–140]: IVIGs (blinded)/plasma exchange (unblinded) were more effective than the placebo in a study of 29 children [137]; in another study, IVIGs were not superior to placebo in a controlled approach in 35 patients [138]. One study found azithromycin slightly superior to placebo in acute OCD in 31 children [139]. In contrast, Snider et al. (2005) found no benefit of azithromycin in 23 children with PANDAS [140]. Despite initial reports of patients who have been successfully treated with antibiotics, anti-inflammatory medication, or immunotherapies (e.g., [67], as summarized in [71], the scientific evidence for the individual treatment approaches is limited thus far, necessitating their systematic investigation in terms of RCTs in patients with PANDAS/PANS.

### Other immunological treatment experiences and considerations for autoimmune OCD

Regarding the treatment of underlying AEs, rheumatic diseases, or neurological diseases such as multiple sclerosis, we refer to the corresponding scientific literature. In these disorders, established immunotherapies such as steroids, plasma exchange, IVIGs, or rituximab are often used [42, 83, 88, 109]. Infectious processes can be treated with antibiotics, antivirals, or antiparasitic substances [141]. In patients with infection-triggered autoimmunity and with primarily autoimmune subtypes, treatment trials with anti-inflammatory and/ or immunotherapies could turn out to be useful [40, 83, 88, 109, 134, 136] (Fig. 3).

## Perspectives and limitations

### Perspectives: the concept of *autoimmune OCD*

The clinical experience with patients with PANDAS/PANS and other autoimmune disorders points to the existence of secondary autoimmune forms of OCD, at least in some patients with atypical clinical manifestations (e.g., [42, 67, 71]). Several studies on different immunological markers support this hypothesis [26]. Especially in the presence of the “red flag” symptoms mentioned in Box 2, an autoimmune etiology should be considered, and extended diagnostic investigations seem to be warranted (see Table 3). It is not yet clear whether a classical primary presentation of OCD excludes secondary causes, which should be investigated in the future. Pathophysiologically, the following subtypes should currently be distinguished:

1. OCD with PANDAS/PANS,
2. OCD with neuronal antibodies: a. against well-characterized cell surface antigens (such as NMDA-R), b. against well-characterized paraneoplastic, intracellular antigens (such as Ma2), and c. against non-well-characterized and novel neuronal autoantibodies,
3. OCD in the context of systemic autoimmune diseases with potential brain involvement (such as systematic lupus erythematosus),
4. OCD in the context of established autoimmune CNS disorders (such as multiple sclerosis).

As a first step towards developing diagnostic criteria, Table 4 presents a preliminary concept for the classification of symptoms and findings in this context operationalizing possible, probable, and definitive autoimmune OCD forms (Table 4). These criteria—inspired by the model of AP [75]—could serve as a basis for further research to be validated and refined.

**Table 4:** Preliminary criteria of possible, probable, and definite autoimmune obsessive-compulsive disorders suggested by the authors.

Possible autoimmune OCD*	Probable autoimmune OCD*	Definite autoimmune OCD*
(Sub)acute onset of OCD symptoms (< 3 months) AND/OR treatment resistance despite guideline-based therapy in combination with at least one of the following signs:	Combination of *possible autoimmune OCD* AND	*Probable autoimmune OCD* (suspected clinical and diagnostic findings) AND
• Atypical age of onset (early childhood or later adulthood)	at least two suspicious alterations in diagnostic investigations:	• Evidence for IgG neuronal antibodies in CSF and/or
• Atypical presentation of obsessive-compulsive symptoms (e.g., combination with severe hypersomnia or loss of function due to disproportionate cognitive deficits)	• Serum: Neuronal autoantibodies, “potentially neuronal” antibodies (e.g., ANAs against dsDNA), streptococcal antibodies	• Successful immunotherapy
• Accompanying neurological signs (movement disorder, focal neurological deficits, new seizures or headache)	• EEG: Signs of encephalopathy such as spike-wave activity or intermittent slowing	
• Autonomic dysfunction	• MRI: Basal ganglia/mesiotemporal hyperintensities, inflammatory lesions	
• Adverse response to antipsychotics (malignant neuroleptic syndrome)	• FDG-PET: Encephalitic patterns with disturbed metabolism in basal-ganglia, cortical or in temporal regions	
• Association of OCD onset with infections	• CSF: CSF-pleocytosis, CSF-specific oligoclonal bands, detection of (neuronal) autoantibodies, increased antibody indices	
• Comorbid autoimmune diseases (with potential brain involvement)		
• Comorbid malignancies		

*ANA* antinuclear antibodies, *OCD* obsessive-compulsive disorder, *dsDNA* double strand deoxyribonucleic acid, *EEG* electroencephalography, *MRI* magnetic resonance imaging, *FDG-PET* [18F]fluorodeoxyglucose positron emission tomography, *CSF* cerebrospinal fluid, *IgG* immunoglobulin G.

The criteria are inspired by the concept of autoimmune psychosis by Pollak et al., 2020 [75]. These criteria should be evaluated and refined over time. *Classification as possible, probable, or definite autoimmune OCD requires exclusion of more likely alternative differential diagnoses (e.g., infectious, metabolic, toxic, “syndromal genetic” forms).

Table 5 provides an overview of the presently suggested syndromal criteria for autoimmune OCD compared with established ICD-10/11 and DSM-5 classification criteria for OCD, as well as PANDAS/PANS criteria. Notably, specific criteria for autoimmune forms of OCD in adults do not exist yet.

**Table 5:** Diagnostic criteria following ICD-10/11 and DSM-5 in comparison with PANDAS/PANS and suggested autoimmune OCD criteria [9,34, https://www.who.int/classifications/icd/en/GRNBOOK.pdf; https://icd.who.int/browse11/l-m/en#/http%3a%2f%2fid.who.int%2ficd%2fentity%2f1126473669].

ICD-10/11 and DSM-5 criteria for OCD	PANDAS criteria	PANS criteria	Suggested autoimmune OCD criteria
• Clinical criteria including psychiatric symptoms.	• Clinical criteria including psychiatric and neurological symptoms.	• Clinical criteria including psychiatric, internal, and neurological symptoms.	• Clinical criteria including psychiatric, internal, and neurological symptoms.
• Established for children and adults.	• Association with a causal factor (streptococcal infection).	• Established only for children.	• Should include laboratory, EEG, and MRI findings.
• Developed for the large group of primary OCD forms.	• Established only for children.	• Developed for the small group of secondary OCD forms.	• Should be validated for children and adults.
• DSM-5 and ICD-11, but not ICD-10 criteria suggest secondary forms of OCD.	• Developed for the small group of secondary OCD forms.		• Developed for the small group of secondary, autoimmune OCD forms.

*ICD* International Statistical Classification of Diseases and Related Health Problems, *DSM* Diagnostic and Statistical Manual of Mental Disorders, *OCD* obsessive-compulsive disorder, *EEG* electroencephalography, *MRI* magnetic resonance imaging.

In the future, the detection of novel neuronal antibodies associated with OCD could play a relevant role in the diagnosis of autoimmune OCD [37, 142], which has proven fruitful in the case of psychoses [112, 143]. Noteworthy, little is known about the link between T cell responses and OCD. This field should be investigated and will likely improve our understanding of the disease. Finally, identified autoimmune OCD forms can have direct implications for the treatment of OCD and require specific treatment regimens beyond standard-of-care treatment (Fig. 3). Based on possible, probable, and definite autoimmune OCD criteria, treatment approaches for autoimmune OCD could be developed based on the diagnostic certainty of an autoimmune cause because of a multimodal diagnostic evaluation (and not only based on the severity of the symptoms, which has been recommended for PANDAS syndrome so far). Depending on the diagnostic certainty, one could then proceed with a treatment using immunotherapies and escalate in analogy to the situation in AE and AP. This could imply, for example, that even a patient with moderate symptoms who meets the criteria for definite autoimmune OCD would receive immunotherapy. The role of the psychiatrists is central in the diagnostics and in evaluating treatment response in this patient group, and concrete treatment approaches should be developed with multidisciplinary consulting teams, including psychiatrists, neurologists, neuroimmunologists, rheumatologists, and infectiologists. Personalized treatment approaches based on clinical criteria for possible, probable, or definite autoimmune OCD could prevent undertreatment and chronification while also avoiding unnecessary immunotherapies that are not without (potentially very serious) side effects and are sometimes requested by worried patients and relatives because of “non-specific” findings – to this extent it is crucial that any proposed criteria are validated vis a vis their sensitivity and specificity in identifying immunotherapy-responsive cases of OCD. The first clinical trials are already investigating the effects of immunotherapies, such as rituximab, in autoimmune OCD subtypes (https://clinicaltrials.gov/ct2/show/NCT04323566). Based on the experience of patients with AE/AP and other neuroimmunological disorders, it can be assumed that immunotherapy in patients with clear autoimmune causes can lead to an improvement in prognosis [42].

### Limitations

The recommendations elaborated here for autoimmune OCD are based on the consensus of emerging clinical evidence and expert experiences but not on systematic randomized studies [109]. Future systematic research is required to validate the clinical predictive value of the proposed model. Similar developments in the research of AP in psychiatry [75] and AE in neurology [73] can serve as a role model. Diagnostic approaches such as the Cunningham panel for antibody testing in patients with PANDAS/PANS are still controversial [117, 144, 145]. The well-characterized neuronal antibodies established in AE have rarely been investigated in the context of OCD. Therefore, antibody testing with established methods such as fixed cell-based assays, live cell-based assays, immunoblots, and/or indirect immunofluorescence on murine brain sections should be addressed in future studies with OCD patients [73, 75, 119–121, 142]. So far, children have accounted for the majority of patients with PANDAS/PANS, whereas only a few cases have been described in adults. RCTs for the effect of anti-inflammatory treatment approaches and immunotherapies for patients with PANDAS/PANS and autoimmune OCDs are necessary [71].

## Conclusions

There is increasing evidence for secondary immune-mediated forms of OCD. The DSM-5 and novel ICD-11 criteria include the category of secondary OCD, without, however, providing guidelines according to which such a diagnosis should be established. In the current paper, the authors have drafted a first proposal of clinical criteria for the definition of secondary autoimmune OCD. Future studies should investigate the prevalence (e.g., by analyzing the rate of neuronal antibodies in patients with OCD), diagnostic regimes (combination and comparison of different diagnostic methods), and optimal therapy of autoimmune OCD, including the development of clear treatment algorithms and clinical guidelines. Recognizing the autoimmune causes of OCD could inform additional therapeutic options for the affected patients to promote treatment response and reduce chronicity.

## Supplementary information

Supplemental Table 1
