# Supplementary material for: Immunological causes of obsessive-compulsive disorder: is it time for the concept of an “autoimmune OCD” subtype?
Source: Transl Psychiatry. 2022 Jan 10;12:5. doi: 10.1038/s41398-021-01700-4 (PMC8744027; doi:10.1038/s41398-021-01700-4)
Supplement: Supplementary file 1 — Supplemental Table 1 [file 41398_2021_1700_MOESM1_ESM.pdf]

SUPPLEMENTAL MATERIAL

**Pathogen detection**	• Throat culture for Group A streptococcal infection, anti-streptolysin O and anti-DNAse B antibodies<br>• Alternatively, serologies or PCR analyses for mycoplasma pneumonia, influenza, Epstein Barr virus, Borrelia burgdorferi, herpes simplex infection or varicella zoster
**Laboratory analyses**	• Differential blood cell count<br>• Erythrocyte sedimentation rate and C-reactive protein<br>• Metabolic parameters<br>• Urine analysis (in case of abnormalities/pyuria: urine culture)<br>• An extended diagnostic includes:<br>➢ Antinuclear antibodies or fluorescent antinuclear antibody, in case of abnormal antinuclear antibodies further clarification of connective tissue disorders should be performed<br>➢ Antiphospholipid antibodies: Anticardiolipin antibody, dilute Russell's viper venom time, b2-glycoprotein I antibodies<br>➢ Ceruloplasmin and 24 urine copper tests in patients with abnormal liver values or Kayser-Fleischer rings
**Additional diagnostics**	• Brain MRI<br>• EEG (ideally including a "sleep EEG")<br>• In case of abnormal findings in MRI/EEG: CSF analysis (including neuronal antibodies)
**Special markers**	• Antibodies against lysoganglioside, tubulin, dopamine D1/D2 receptor<br>• Antibody produced activation of calcium calmodulin protein kinase II

Supplemental Table 1: Diagnostic examinations in patients with suspected PANDAS/PANS [Chang et al., 2015]. Abbreviations: CSF, cerebrospinal fluid; EEG, electroencephalography; MRI, magnetic resonance imaging; PCR, polymerase chain reaction.

Reference:

Chang et al. PANS Collaborative Consortium. Clinical evaluation of youth with pediatric acute-onset neuropsychiatric syndrome (PANS): recommendations from the 2013 PANS Consensus Conference. J Child Adolesc Psychopharmacol. 2015 Feb;25(1):3-13. doi: 10.1089/cap.2014.0084. Epub 2014 Oct 17.
